# Elbow trauma in children: development and evaluation of radiological artificial intelligence models

**DOI:** 10.1016/j.redii.2023.100029

**Published:** 2023-04-29

**Authors:** Clémence ROZWAG, Franck VALENTINI, Anne COTTEN, Xavier DEMONDION, Philippe PREUX, Thibaut JACQUES

**Affiliations:** 1 Université de Lille , Lille, France; 2 Centre hospitalier universitaire de Lille, Lille, France; 3 Inria Lille – Nord Europe, équipe Scool, Lille, France; 4 CNRS UMR 9189 – CRIStAL, Lille, France; 5 École Centrale de Lille, Lille, France

**Keywords:** X-ray, Elbow, Pediatrics, Convolutional neural networks (CNN), Deep learning

## Abstract

**Rationale and Objectives:**

To develop a model using artificial intelligence (A.I.) able to detect post-traumatic injuries on pediatric elbow X-rays then to evaluate its performances in silico and its impact on radiologists’ interpretation in clinical practice.

**Material and Methods:**

A total of 1956 pediatric elbow radiographs performed following a trauma were retrospectively collected from 935 patients aged between 0 and 18 years. Deep convolutional neural networks were trained on these X-rays. The two best models were selected then evaluated on an external test set involving 120 patients, whose X-rays were performed on a different radiological equipment in another time period. Eight radiologists interpreted this external test set without then with the help of the A.I. models .

**Results:**

Two models stood out: model 1 had an accuracy of 95.8% and an AUROC of 0.983 and model 2 had an accuracy of 90.5% and an AUROC of 0.975. On the external test set, model 1 kept a good accuracy of 82.5% and AUROC of 0.916 while model 2 had a loss of accuracy down to 69.2% and of AUROC to 0.793. Model 1 significantly improved radiologist's sensitivity (0.82 to 0.88, *P* = 0.016) and accuracy (0.86 to 0.88, *P* = 0,047) while model 2 significantly decreased specificity of readers (0.86 to 0.83, *P* = 0.031).

**Conclusion:**

End-to-end development of a deep learning model to assess post-traumatic injuries on elbow X-ray in children was feasible and showed that models with close metrics in silico can unpredictably lead radiologists to either improve or lower their performances in clinical settings.

## 1 Introduction

Detection of fractures is an issue in musculoskeletal imaging. Indeed, missed fractures represent more than 80% of diagnostic errors in the emergency department (ED) [1,2].

Trauma of the upper limb is a frequent reason for consultation in the ED and generally the clinical examination guides the indication for radiographic workup [3,4]. Misdiagnosis is more common in radiographic children due to the lack of ossification centers, particularly considering the elbows [5,6]. Thus, the highest rate of diagnostic error in upper limb fractures in children lays at the elbow (77% of diagnostic errors) [7,8]. Despite the expertise required to interpret an X-ray of the child's elbow, it is pediatricians (and not radiologists) that read radiographs in the first line in many pediatric emergency departments. The issue of access to pediatric imaging radiologists is a problem in many centers and artificial intelligence could be used as a diagnostic aid.

Several studies have shown the usability of deep convolutional neural networks (CNNs) in fracture detection on radiographs [9], [10], [11], [12], [13]. However, most of the artificial intelligence (A.I.) models that can detect fractures on X-ray are not validated in children, except recently [14], [15], [16], [17]. Moreover, despite numerous publications, focus is mostly made on the in silico performance of algorithms, but sparsely on the performance resulting from the interaction between humans and A.I. [11,18,19]. In addition, recent work on this topic tends to show the potentially deleterious impact of a model, though effective in silico, on the diagnoses made by doctors [20,21].

The purpose of this study was therefore to develop a model using artificial intelligence to detect post-traumatic injuries on pediatric elbow X-rays, and then to assess the impact of its use on radiologists’ performances.

## 2 Material and methods

### 2.1 Data collection

All elbow X-rays performed in patients aged between 0 and 18 years in the event of an acute trauma of the upper limb, between January 1, 2015 and August 31, 2019 in the emergency department were retrospectively gathered from the PACS (Intellispace PACS, Philips). All series were kept, including those with only one view, more than two views and sub-optimal views, because of their frequency in children, especially after an upper limb trauma. A total of 1956 X-rays from 935 patients (485 male and 450 female) were collected and de-identified. The de-identification of data and their retrospective analysis was approved by the institutional board under the reference DEC19-279. All these examinations were performed on the same X-ray device (Fujifilm, Tokyo, JP).

**Fig. 1:**
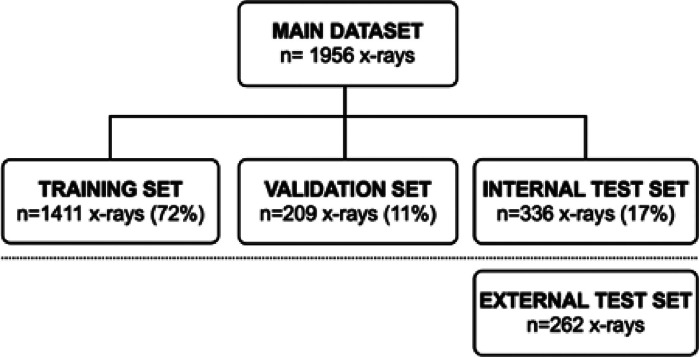
Repartition of the datasets. n: number of radiographs.

Radiographs were randomly divided into a training set (668 patients, average age 10), a validation set (99 patients, average age 10) and an internal test set (168 patients, average age 9). (Fig. 1)

External test set was composed of 262 elbow radiographs performed in 120 patients (average age 10), on another X-ray device (General Electrics, USA) and in another time period (July to December, 2014).

### 2.2 Reference standard and labelling

Two radiologists (with 2 and 7 years of experience in pediatric trauma radiology) classified in consensus all the elbow X-rays (internal and external test set) in two groups: normal or abnormal. Examinations were labelled as abnormal in the following situations: visible fracture, articular dislocation, soft tissues change of potential post-traumatic origin (e.g. fat pad sign, even if no fracture was directly visible). A total of 1171 radiographs were considered normal and 785 abnormal (Table 1).

**Table 1:** Repartition of examinations in the different datasets depending on the label. *N*: number of radiographs.

	Pathological findings, *N* (%)	Normal examinations, *N* (%)
Training set (*N* = 1411)	537 (38%)	874 (62%)
Validation set (*N* = 209)	92 (44%)	117 (56%)
Test set (*N* = 336)	156 (47%)	180 (53%)

### 2.3 Training of the model

The training and validation sets were used to optimize deep convolutional neural network (CNN) by modulating architecture, depth and hyperparameters. Thousands of models have been trained following a gradient descent algorithm and by varying four main hyperparameters: model architecture, patience, learning rate and image dimensions.

### 2.4 Image dimension

Originally, DICOM files had a dimension of 4096×4096 pixels. To reduce the computational cost of training the CNN, this resolution was downscaled to 1024×1024 for each radiograph after a cheking for labelling transfer. Then, radiologists identified an area containing the whole elbow joint on each image, using a generic labelling tool (RectLabel, Mac Os) in order to obtain cropped images (Crop 224 × 224). To obtain Crop 512 × 512 X-rays, the original X-ray was downscaled to 2048 × 2048 and the bounding box from Crop 224 × 224 was converted on this image in order to obtain the same area but with higher precision.

### 2.5 Data augmentation

In order to increase the number of data, vertical and horizontal transformations together with image rotation between -45° and + 45 ° were randomly applied. The image normalization was performed using conventional ImageNet normalizations since the CNN was pretrained using ImageNet.

### 2.6 Visualisation tool

Grad-CAM algorithm was implemented to visualize the class activation heatmap of the algorithm. The scale of the Grad-CAM varied from blue (low) to red (high) depending on the weight of each area in the final output of the algorithm.

### 2.7 Internal and external validation

Performances of models were measured on the internal test set which was composed of 336 X-rays, used neither in training nor in validation sets. The two best models regarding accuracy and area under the receiver-operator curve (AUROC) were kept and called model 1 (M1) and model 2 (M2). Sensitivity, specificity, Youden index and accuracy were calculated. Then, performances of these two best models were also measured on external test set.

### 2.8 Evaluation of radiologists

Eight radiologists were included in this analysis, with an experience in trauma radiology ranging from 6 months to 10 years: 4 radiology residents and 4 seniors radiologists specialized in musculoskeletal imaging.

All radiologists analysed the 120 studies (262 X-rays) of the external test set during two sessions, blinded from the other readers, and without time limitation during the session.

During these two sessions, radiologists interpreted all radiographs without then with the help of A.I.. All readers used one model during each session, M1 or M2. Model allocation was distributed randomly during the first session. After a wash-out time of two months, during the second session, radiologists performed a new reading session, using the other model.

The deidentified examinations were interpreted on their usual workstations, without access to previous or follow-up examinations to avoid follow-up bias. Radiologists were asked to first classify the study in being either normal or abnormal, prior to getting A.I. results, then to provide their final opinion after getting A.I. results.

Intra-reader agreement was measured between the two sessions for each radiologist. The sensitivity, specificity, accuracy and Youden index were calculated for each radiologist, before and after the use of A.I..

### 2.9 Statistical analysis

Analyses were performed using Prism 9 software (GraphPad, La Jolla, CA). Quantitative data were reported as mean±standard deviation. Qualitative data were reported as raw number and percentage (%). The significance threshold was set at *P* < 0.05. To compare radiologists’ performances before and after the use of A.I., Wilcoxon matched-pairs signed ranked test was used for sensitivity, specificity, accuracy and Youden index analysis.

## 3 Results

### 3.1 Internal validation

On the 168 patients (336 X-rays) from the internal test set, 78 had at least one abnormal finding, while 90 studies were free of post-traumatic findings.

**Fig. 2:**
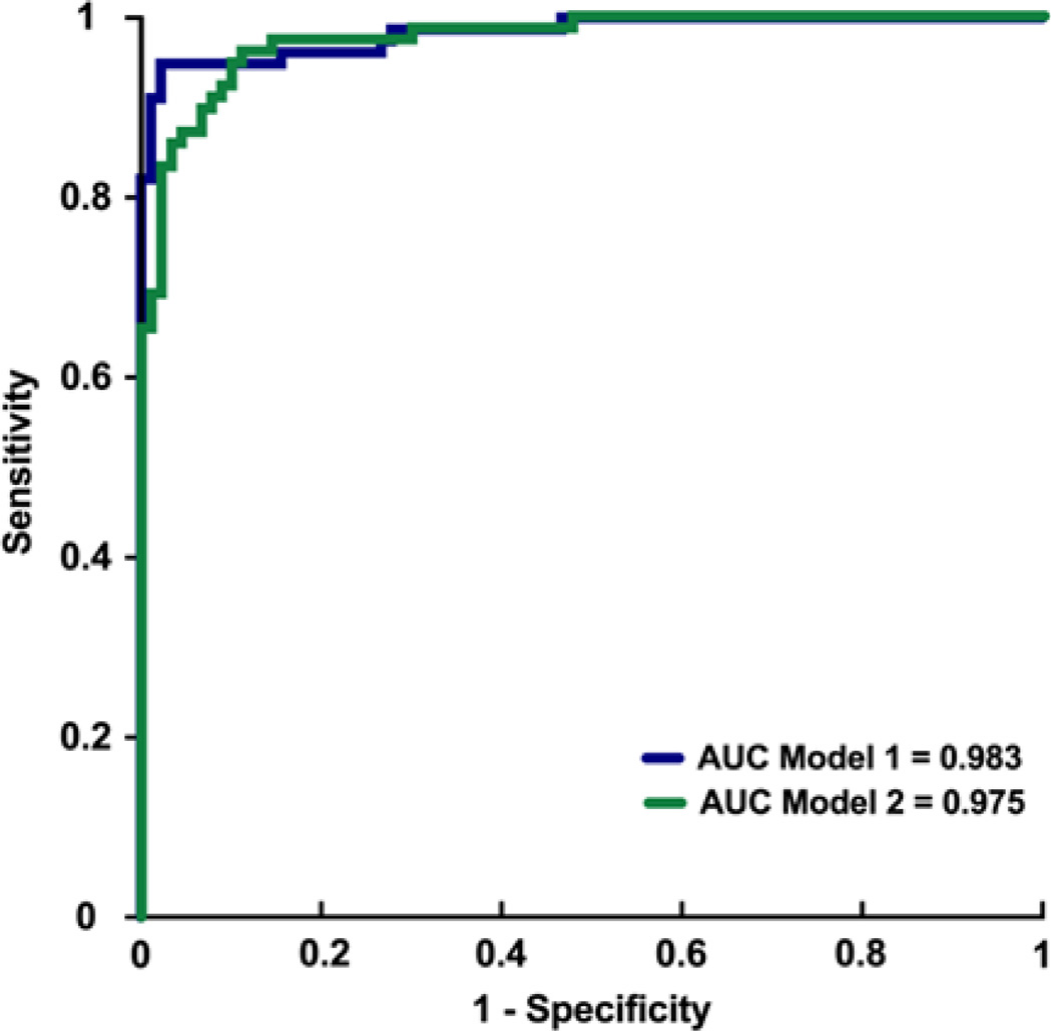
Receiver-operator characteristic (ROC) curves of the two best models on internal test set. AUC: area under the ROC curve.

Two models using deep convolutional neural networks stood out. these two models had similar values ​​for patience and learning rate. The best model, model 1 (M1), used cropped 512 × 512 images whereas the second best model called model 2 (M2) used 512 × 512 uncropped images. M1 showed the higher AUROC (0.983) and the best compromise between sensitivity (0.935) and specificity (0.978), with an accuracy of 95.83%. M2 used 512 × 512 uncropped images and presented an accuracy of 90.48%, an AUROC of 0.975, a sensitivity higher than M1 (0.974) but a lower specificity (0.844). Performances of the models on the internal test set are reported in Table 2 and their receiver-operator curves (ROC) are showed in Fig. 2.

**Table 2:** Evaluation of the two best models on the internal test set. AUROC: area under the ROC curve.

	Model 1 (M1)	Model 2 (M2)
AUROC	0.983	0.975
Accuracy	0.958	0.905
Sensitivity	0.935	0.974
Specificity	0.978	0.844
Youden index	0.913	0.818

### 3.2 External validation

The external test set was composed of 61 patients with normal examinations, and 59 patients with at least one abnormal finding on X-ray. The performances of M1 and M2 on this dataset are reported in Table 3.

**Table 3:** Evaluation of M1 and M2 on the external test set. AUROC: area under the ROC curve.

	Model 1 (M1)	Model 2 (M2)
AUROC	0.916	0.793
Accuracy	0.825	0.692
Sensitivity	0.847	0.915
Specificity	0.803	0.475
Youden index	0.648	0.389
Contingency table for model 1:		
	Fract	Normal X-Ray
Positive test	TP = 50	FP = 12
Negative test	FN = 9	VN = 49
Contingency table for model 2:		
	Fract	Normal X-Ray
Positive test	TP = 54	FP = 32
Negative test	FN = 5	VN = 29

**Fig. 3:**
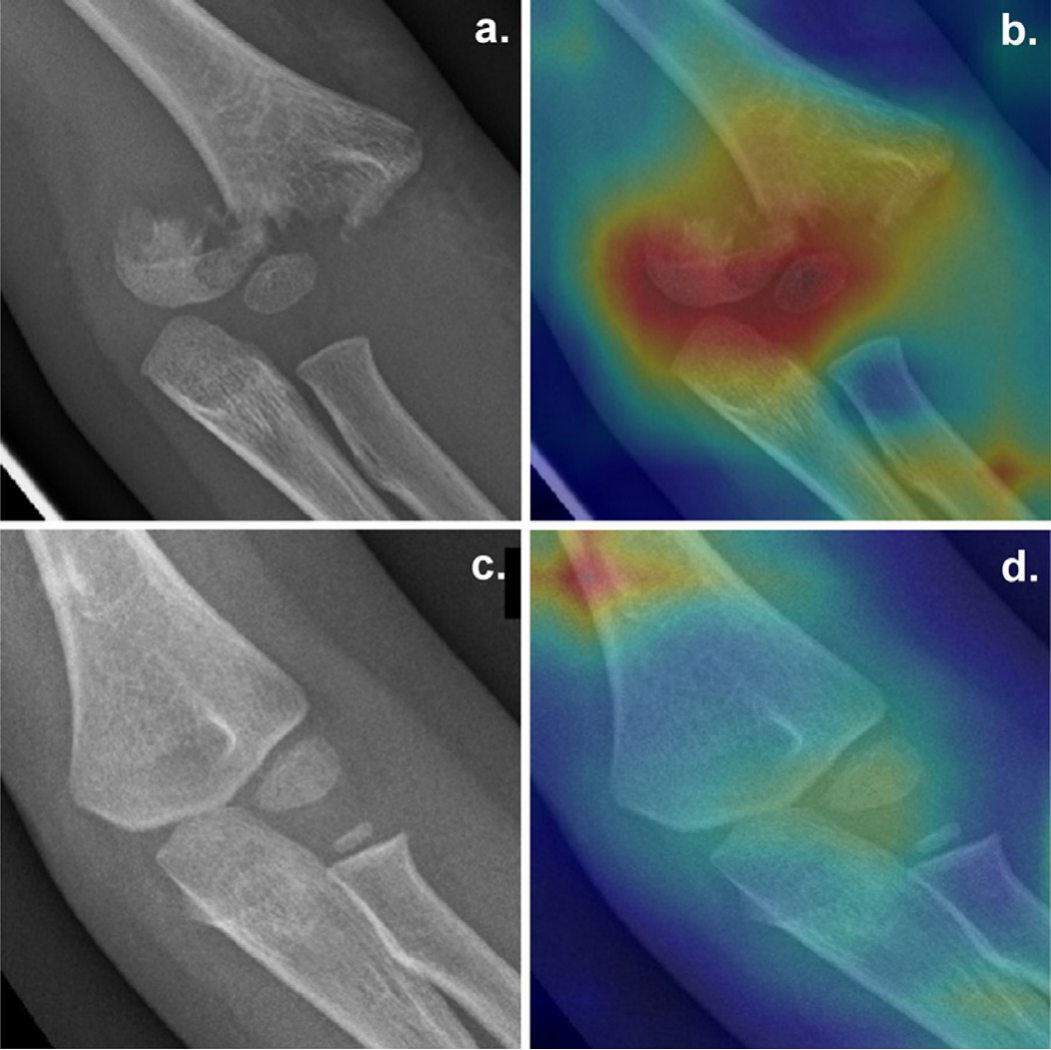
a,b: Example of true-positive Grad-CAM with pallet humeral frontal X-ray in a 4 year old boy and corresponding Grad-CAM using M1; c,d: Example of false-positive case: supracondylar process which was inadequately considered as a fracture by the model in a 6-year old girl using M1.

While M1 showed a moderate change in AUROC values (0.983 to 0.916), M2 showed a significant drop in AUROC between internal and external test sets (0.975 to 0.793), mostly due to a significant drop in specificity (0.844 to 0.475). Fig. 3 shows heatmaps after using M1.

### 3.3 Interaction with radiologists

Intra-reader agreement of examinations without A.I. between both sessions was excellent: 0.92 ± 0.021 for all radiologists (*N* = 8), 0.92 ± 0.02 for radiology residents (*N* = 4) and 0.91 ± 0.02 for senior radiologists (*N* = 4).

**Fig. 4:**
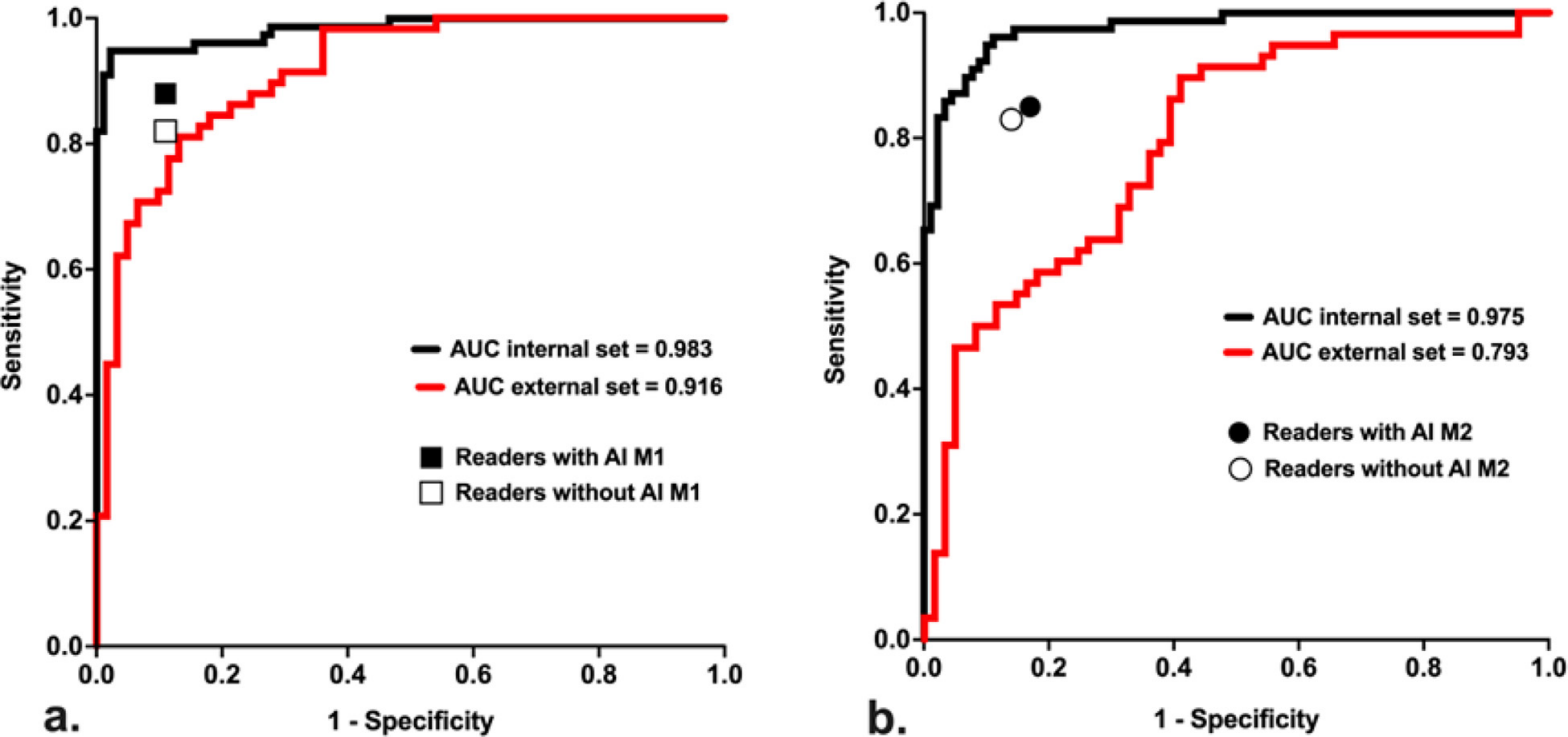
Variation of radiologists' sensitivity and specificity with the use of A.I. models (M1 (a) or M2 (b)), plotted adjacent to ROC curves of both models (on the internal test set in black and on the external test set in red). AI: artificial intelligence; M1: model 1; M2: model 2; AUC: area under the curve.

Radiologists’ performances without and with A.I.-models are reported in Table 4, and represented in Fig. 4.

**Table 4:** Radiologists’ sensitivity, specificity, accuracy and Youden index before and after the use of model 1 (M1) or model 2 (M2). SD: standard deviation.

	Before M1	After M1	*P*	Before M2	After M2	*P*
Sensitivity {SD}	0.82	0.88 {0.01}	0.02 (*)	0.83	0.85 {0.04}	0.06
Specificity {SD}	0.89	0.89 {0.30}	0.38	0.86	0.83 {0.01}	0.03 (*)
Accuracy {SD}	0.86	0.88 {0.06}	0.047 (*)	0.85	0.84 {0.53}	0.75
Youden {SD}	0.71	0.76 {0.05}	0.04 (*)	0.69	0.69 {0.62}	0.94

As showed in Table 4 and Fig. 4, model 1 significantly improved radiologists’ sensitivity (*P* = 0.016), accuracy (*P* = 0.047) and Youden index (*P* = 0.039), while model 2 significantly decreased specificity of readers (*P* = 0.031).

As shown in Fig. 4, although the initial performance of radiologists was superior to the models on the external test set, reader performances still improved significantly with the help of model 1, while model 2 did not improve the performances of readers and even significantly reduced their specificity.

**Fig. 5:**
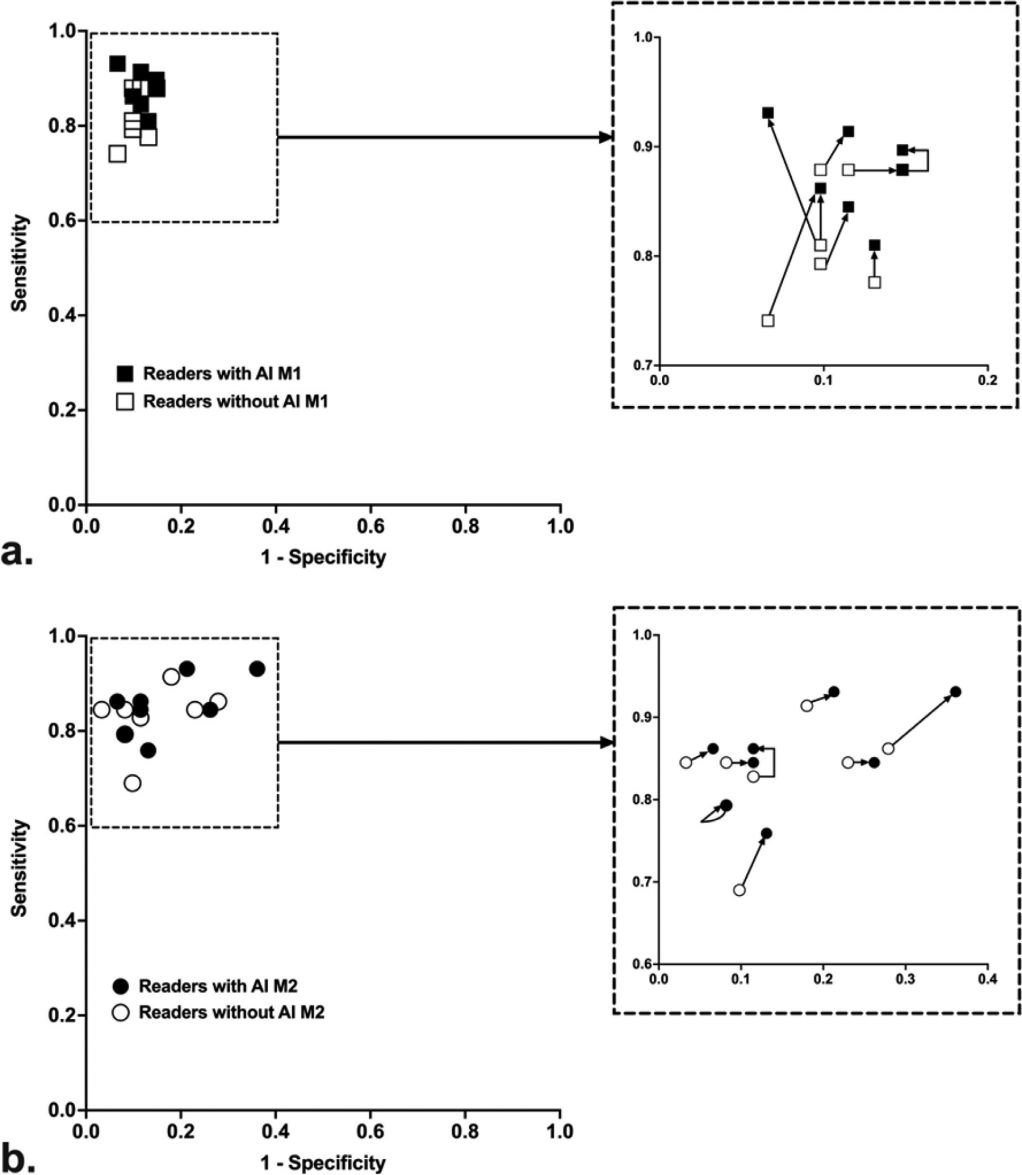
Plot of all readers without then with the use of model 1 (a) or model 2 (b). AI: artficial intelligence; M1: model 1; M2: model 2.

As shown in Fig. 5, concerning model 1, the majority of readers (*N* = 7) increased their sensitivity but to the detriment of a slight drop in specificity for half of them (*N* = 4). One radiologist lowered his specificity (from 0.89 to 0.85) without changing his sensitivity (0.88), therefore displaying lower performances with the help of the model.

Concerning model 2, more than half of radiologists (*N* = 6) reduced their specificity, while the other (*N* = 2) displayed no change in specificity. No reader had an improved specificity with the use of M2.

**Fig. 6:**
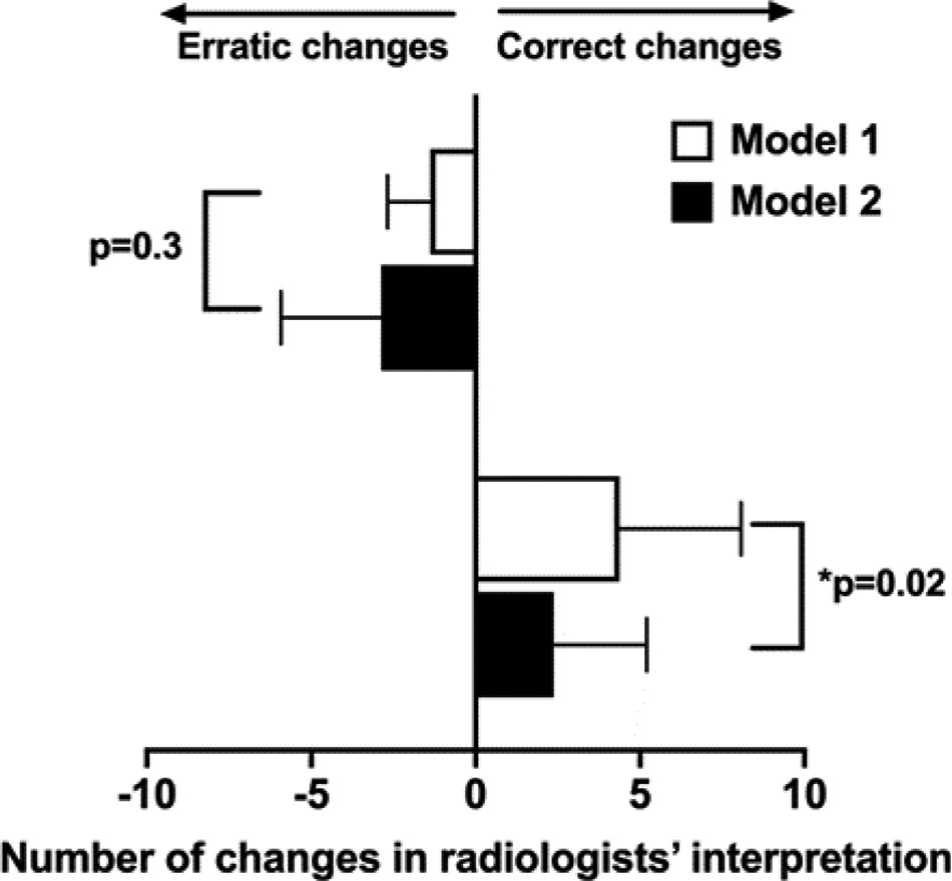
Correct or erratic changes in radiologist's interpretation after the use of A.I., for each model.

Regarding practical changes in radiologists’ interpretation, Fig. 6 summarizes the mean number of erratic changes (i.e. radiologists being correct before A.I. but incorrect after A.I.) and correct changes (i.e. radiologists being incorrect before A.I. but correct after A.I.), for each model. M1 led to a significantly higher number of correct changes among radiologists compared to the use of M2: 4.4 ± 3.7 with M1 as compared to 2.4 ± 2.8 for M2 (*P* = 0.02).

Finally, when considering the balance of positive changes for each radiologist (number of correct changes – number of erratic changes / total number of cases), M1 displayed a positive balance of +2.2% ± 2.7% and M2 a significantly negative balance of -0.42% ± 1.4 (*P* = 0.047).

## 4 Discussion

This study showed that an A.I. model using a CNN architecture can detect post-traumatic injury on pediatric elbow X-rays. AUROC values on the internal test set were high for both M1 and M2 (0.983 and 0.975 respectively), which is better than a lot of artificial intelligence publications on conventional X-rays so far [22].

There are real strengths of this study. First of all, we have included all the X-rays, even if the quality criteria are not met. Moreover, training and external test were realized on two different X-ray devices. Indeed, the external test were performed on X-rays realized with another device and another period. In most of the cases of nondisplaced fracture, the diagnoses relied only on the presence of the fat pad displacement. With model 1 and 2, using respectively cropped and uncropped X-rays, only 9 and 5 respectively non displaced fracture were missed (False negative).

Both models showed a drop in AUROC on the external test set (0.916 and 0.793 respectively), which is consistent with a tendency of CNN-based models to overfit on internal test sets [23]. However, the higher drop of M2 on the external test set raised concerns about its potential generalizability and showed that two models that display close AUROC values on internal data can undergo significantly different changes when exposed to another dataset as demonstrated by Wang et al. on mammogram classification [24]. The magnitude of these changes is unpredictable as Voter et al. showed in their study [25]

Radiologists’ performances were overall lower than the internal performances of both M1 and M2 in silico. However, even without the help of A.I., radiologists’ performances were superior to those of both M1 and M2 on external test sets, which stresses out that comparison between human and algorithms on the sole internal test sets should be avoided, since they tend to overclaim the inner performances of the algorithms.

Although the initial performances of radiologists were superior to those of both models on the external test set, human performances still improved significantly with the help of model 1. On the contrary, model 2 did not improve the performance of readers and even significantly reduced their specificity. Model 2, which was supposedly the most sensitive model based on internal test set values, tended to mislead radiologists in their interpretation. These findings are crucial since they show that the actual impact a model can have on humans is difficult to precisely appreciate beforehand. The performances of M1 being slightly inferior to humans on the external test set could have implied that M1 cannot actually improve their performances, while it actually did. On the other hand, the high sensitivity of M2 in silico could have implied that the model would increase readers’ performances, while it in fact misled them more often.

Moreover, consequences of both algorithms on the radiologists’ decision were measured (i.e. changes in individual interpretation after the use of A.I.). When considering practical impact on a population of patients of the use of A.I. by a radiologist, the key question would be the actual balance between the number of cases where changes in interpretation would benefit the patient (correct changes) and changes that could impair the patient (erratic changes). Our results showed that there was a significant difference in the final benefit, since the use of M1 resulted in an average gain of +2.2% in correct changes, while the use of M2 would result in a negative balance (-0.42% of correct changes). However, the magnitude of these changes remains low.

There are some limitations in our study. First of all, though pretrained on ImageNet, our model was developed on a dataset of relatively limited size. Indeed, many algorithms focusing on conventional X-rays rely on larger datasets [10]. Nevertheless, few have focused on the specific condition of elbow trauma in children, due to the lower availability of such data as compared to those in adults [26]. To compensate for the size of the dataset, conventional techniques of data augmentation were performed, but are weaker to prevent overfitting as compared to new data, which can partly explain the changes observed between internal and external test sets [27]. Secondly, this study was monocentric and showed that results can be variable when exposing algorithms on an external test set (on another device). This stresses out the urge for multicentric trials in the field of A.I. in radiology. Finally, the number of readers in this study was limited (*N* = 8), though higher than in several publications [28,29], which did not enable to display differences between junior and senior readers. Further studies with more readers of different profiles are needed to confirm these results and better understand the relations between algorithms outputs and human performances. Indeed, in many pediatric emergency departments, it is pediatricians (and not radiologists) that read radiographs in the first line. It would be interesting to evaluate the positive or negative impact of A.I. models on them.

## 5 Conclusion

End-to-end development of a CNN model to assess post-traumatic injuries on elbow X-ray in children was feasible. Models with close metrics in silico can unpredictably lead radiologists to either improve (M1) or lower (M2) their performances in clinical settings, underlining the need for further precise clinical evaluation of A.I.-based tools.

## Declaration of Competing Interest

The authors declare that they have no known competing financial interests or personal relationships that could have appeared to influence the work reported in this paper.
